# TRACK—a new algorithm and open-source tool for the analysis of pursuit-tracking sensorimotor integration processes

**DOI:** 10.3758/s13428-023-02065-w

**Published:** 2023-01-25

**Authors:** Adriana Böttcher, Nico Adelhöfer, Saskia Wilken, Markus Raab, Sven Hoffmann, Christian Beste

**Affiliations:** 1 Department of Child and Adolescent Psychiatry, Faculty of Medicine, Cognitive Neurophysiology, TU Dresden, Fetscherstraße 74, 01307 Dresden, Germany; 2 Faculty of Medicine, University Neuropsychology Center, TU Dresden, Dresden, Germany; 3 General Psychology: Judgment, Decision Making, & Action, Institute of Psychology, University of Hagen, Hagen, Germany; 4 Performance Psychology, Institute of Psychology, German Sport University Cologne, Cologne, Germany; 5 School of Applied Sciences, London South Bank University, London, UK

**Keywords:** Sensorimotor integration, Tracking task, Algorithm

## Abstract

In daily life, sensorimotor integration processes are fundamental for many cognitive operations. The pursuit-tracking paradigm is an ecological and valid paradigm to examine sensorimotor integration processes in a more complex environment than many established tasks that assess simple motor responses. However, the analysis of pursuit-tracking performance is complicated, and parameters quantified to examine performance are sometimes ambiguous regarding their interpretation. We introduce an open-source algorithm (TRACK) to calculate a new tracking error metric, the spatial error, based on the identification of the intended target position for the respective cursor position. The identification is based on assigning cursor and target direction changes to each other as key events, based on the assumptions of similarity and proximity. By applying our algorithm to pursuit-tracking data, beyond replication of known effects such as learning or practice effects, we show a higher precision of the spatial tracking error, i.e., it fits our behavioral data better than the temporal tracking error and thus provides new insights and parameters for the investigation of pursuit-tracking behavior. Our work provides an important step towards fully utilizing the potential of pursuit-tracking tasks for research on sensorimotor integration processes.

## Introduction

Sensorimotor integration processes are the fundament of many cognitive functions in everyday life and are at the center of many cognitive (neuro)science theories on how the integration of perceptual and motor processes unfolds (Hommel et al., 2001). In recent years, these processes have been subject to many studies investigating the learning and execution of complex motor skills, their influencing factors and transfer to related movements such as in driving (e.g., Broeker, Haeger, et al., 2020b). The exact mechanisms behind such sensorimotor integration processes have been the subject of different theories over the years. For instance, the perceptual control theory (PCT; Powers, 1973) established motor control as the control of sensory input via negative feedback loops (Marken et al., 2013). In the framework of this theory, perceptual input generated by motor processes is hierarchically controlled depending on the level of perception. The input is compared to a reference variable that is set endogenously and the motor output is adjusted accordingly (Parker et al., 2020). The type of perceptual input, such as a moving target, thereby imposes temporal constraints on motor control processes (Marken et al., 2013). Sensorimotor integration processes thus depend on the level of perceptual input, with more effort needed for the perception of complex stimuli as in everyday life. PCT-based models were able to simulate and predict individuals’ performance in a pursuit-tracking paradigm (Parker et al., 2017). Along these lines the field has also moved to more computational accounts of how sensorimotor integration processes are accomplished (Franklin & Wolpert, 2011; Körding & Wolpert, 2004) and how such processes can contribute to aspects of cognitive control and intentions to act (Christensen & Bicknell, 2022).

Crucially, regardless of the conceptual background, most experimental paradigms assessing cognitive and motor functions related to sensorimotor integration use behavioral readouts such as button presses. These are unlikely to operationalize complex sensorimotor processes, thus indicating a need for paradigms putting higher demands on sensorimotor integration processes with higher ecological validity (Hill & Raab, 2005; Hoffmann et al., 2018; Wulf & Shea, 2002).

The pursuit-tracking paradigm offers a continuous and complex experimental setting used to examine sensorimotor integration processes in more ecologically valid settings (Broeker, Ewolds, et al., 2020a; Broeker, Haeger, et al., 2020b; Hill, 2009, 2014; Hill & Raab, 2005; Parker et al., 2020; Pew, 1974; Sekiya, 2006; Wulf & Schmidt, 1997). In a typical pursuit-tracking task, participants watch a target moving along an (invisible) trajectory. The trajectory is usually a horizontally oriented waveform built using randomly generated sine and cosine waves (Wulf & Schmidt, 1997; see Methods section). Often, the first and last parts of the waveform are newly generated for each trial (i.e., the coefficients for the sine and cosine are randomly selected), whereas other parts of the trajectory remain constant across all trials (e.g., Künzell et al., 2016) and in some studies also across participants (e.g., Hill & Raab, 2005; Raab et al., 2013; Wulf & Schmidt, 1997). The pursuit-tracking task serves as a tool to investigate online sensorimotor integration processes by providing continuous data of participants trying to follow a steadily moving target. The pursuit-tracking paradigm thus enables a continuous measure of the quality of behavioral adaptation processes. This sharply contrasts with experimental operationalizations commonly used to examine behavioral adaptation processes in which behavioral adjustments occur discretely in time (e.g., in Flanker, Stroop, Simon or other tasks in which behavioral adaption is measured across discrete trials via distinct sequences of button press events).

However, to fully utilize the advantage of continuous (real-time) access to behavioral adaptation processes offered by tracking procedures, appropriate behavioral research methods and parameters must be in place to quantify real-time behavioral adaptation or the deviation between the desired and the achieved sensorimotor trajectory. In particular, the choice of a useful metric to access continuous behavioral adjustments crucially depends on the device operated by the participants. Pursuit-tracking tasks are often executed using a joystick (Broeker et al., 2021; Broeker, Ewolds, et al., 2020a; Broeker, Haeger, et al., 2020b; Ewolds et al., 2017, 2021; Hill, 2014; Künzell et al., 2016). Importantly, in such studies, participants can usually only control the vertical movement of the cursor. Often, the horizontal motion is fixed to the target (i.e., x-axis speed of target and cursor are aligned) to prevent participants from cutting trials short by moving the cursor straight to the aimed edge of the screen (Broeker, Ewolds, et al., 2020a; Broeker, Haeger, et al., 2020b). In contrast, in studies using other devices, such as a computer mouse, participants can often control the horizontal as well as vertical movement of the cursor (Hill, 2014; Raab et al., 2013; Zhu et al., 2014). Here, the task has a different data output since the difference between cursor and target positions can be analyzed in two dimensions instead of one. However, these differences in the data structure are mostly not accounted for in analyzing the tracking data. Commonly, data analysis is based on calculating the root-mean-square error (RMSE), summing the distance between cursor and target at each time point. While this seems reasonable for data generated by participants actively controlling the horizontal and vertical cursor motion, the method has considerable weakness when applied to data that were obtained by participants having only vertical control of the cursor position:

The underlying assumption of the RMSE method is that the target trajectory and pursuit are best matched by comparing the data points measured simultaneously. Following this assumption, a minimum of the error curve, i.e., the minimal distance between target and cursor at time point *t*, occurs when target and cursor cross each other but may move in different directions (see Methods section, Fig. 2). Therefore, this event does not necessarily indicate a peak in tracking performance since it could also be caused by an unexpected change in the target direction. Furthermore, a maximum of this error curve occurs when there is a local maximum of the distance between cursor and target at time point *t*. At this point, it has to be considered that an error maximum can be caused by different processes. For example, an unexpected change of the target direction while the cursor direction remains the same maximizes the distance between the cursor and target. Another potential cause for the sudden maximal distance between target and cursor could be that the direction of the cursor is changed anticipatorily, but there is no equivalent direction change of the target, which would thus be classified as false tracking. The numerous causes for minima and maxima of the error curve led to problems interpreting results based on this method. The often-used averaging of the deviation of target and cursor through the RMSE might be biased by extreme values caused by unexpected trajectory changes rather than by ‘extremely’ false tracking. Thus, another weakness of the simple error curve is that the resulting signal can be intermingled with different cognitive processes, which hinders the interpretability of summed measures such as the RMSE. For instance, continuous tracking processes have been characterized as a complex of sensorimotor feedback loops and feedforward processes, as well as the activation of inner representations of trajectories (Pew, 1974). Consequently, neurophysiological insights into continuous tracking performance are subject to ambiguity. While in some studies this way of analyzing the tracking data is sufficient to detect global differences in performance, such as the enhanced tracking performance during the repeated trajectory part that is induced by implicit learning (Ewolds et al., 2021; Künzell et al., 2016; Wulf & Schmidt, 1997) or related to higher predictability of the target trajectory (Broeker et al., 2021), the increase in the tracking error with time spent on task (Zhu et al., 2014) or the increased tracking performance if there is prior knowledge about the repeated trajectory (Broeker, Ewolds, et al., 2020a). In sum, the RMSE does not fully exhaust the potential and complexity of the data.

The open-source tool and algorithm we present in this study (https://osf.io/stz7u/) foregoes the weaknesses mentioned above and allows a more differentiated analysis of the data, considering the complexity of the pursuit-tracking paradigm. Given that the cursor movement and target trajectory are time series related to each other, we expect that there are (i) segments of anticipation (i.e., there is a cursor direction change that occurs before a target direction change, indicating feedforward processes) and (ii) segments of adaptation (i.e., target direction changes are followed by cursor direction changes, indicating feedback processes). Our method provides a way of calculating the distance between pursuit and aimed target trajectory, thus producing continuous data of the deviation of target and pursuit, which is not prone to suggest random crossings of the cursor and target trajectories as minimal tracking error, which can cause interpretative ambiguities of pursuit-tracking data. For pursuit-tracking data that vary only in the vertical dimension, we propose to distinguish two error types: the *temporal tracking error*, i.e., the simple error curve, and the *spatial tracking error.* We present the algorithm used to calculate the spatial error and apply our method to a sample of pursuit-tracking data (of course the temporal tracking error can also be calculated using the tool). For this sample, we contrast the results for both error types. The code used to implement the presented spatial error algorithm is provided in this publication and can be used for all data generated by the pursuit-tracking task with cursor movements locked to target movements on the x-axis. Furthermore, we provide tracking data of our pursuit-tracking paradigm for two participants (10 trials each) of our empirical sample.

## Methods

### Tracking trajectories

**Fig. 1:**
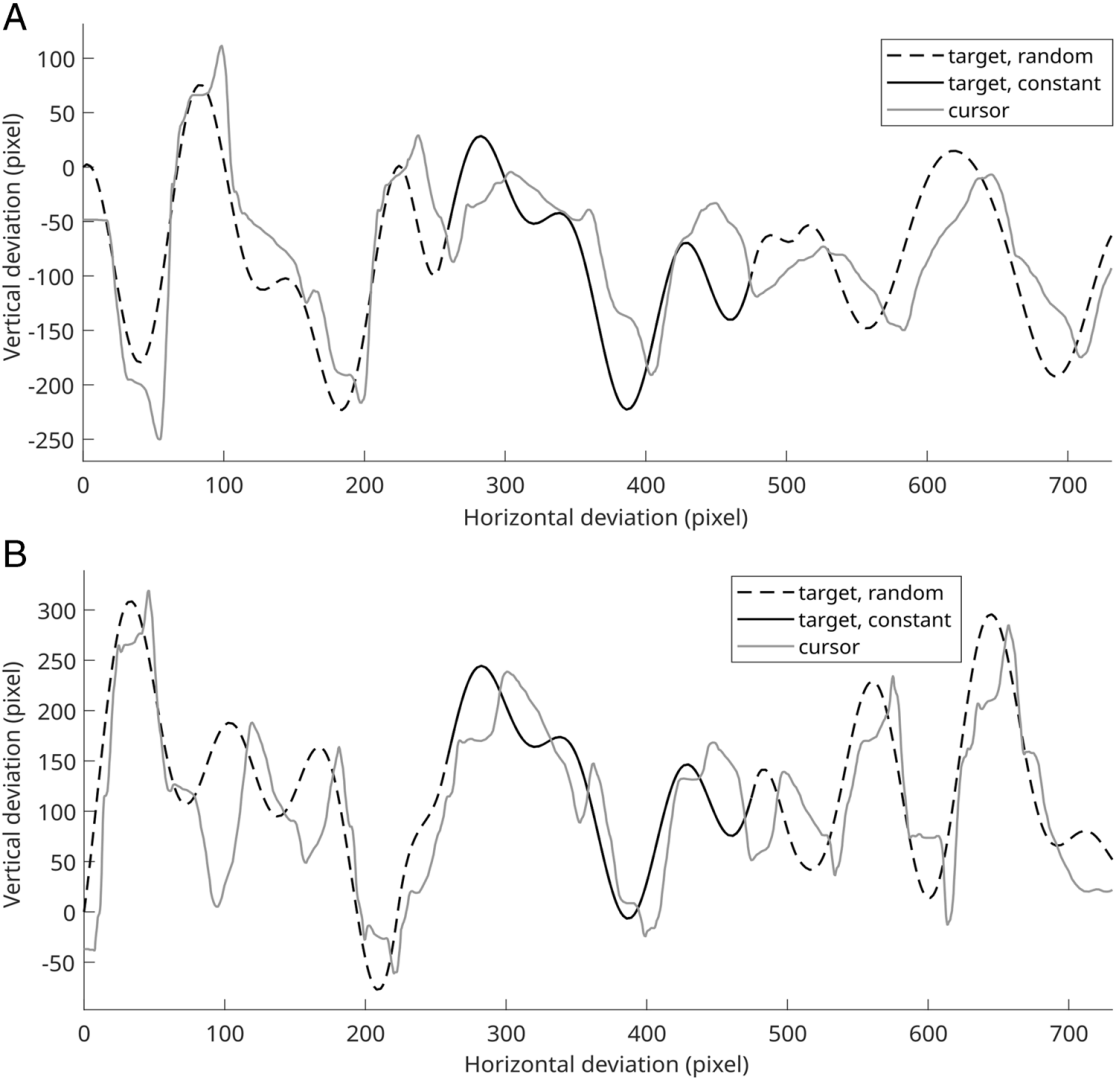
Concatenated target trajectory for two exemplary trials from different participants

The target trajectory consisted of three segments. Each of these segments was created through a concatenation of three sine and cosine terms, by following the formula proposed by Wulf and Schmidt (1997):

$$f(x)=\sum_{i=1}^3{a}_i\bullet \sin \left(i\bullet x\right)+{b}_i\bullet \cos \left(i\bullet x\right)$$

with the coefficients *a* and *b* being randomly generated numbers in the range of −40 and 40 pixels. These boundaries were chosen to ensure that the trajectory stretches to the upper and lower edges of the monitor without transcending them. Importantly, for each target trajectory’s middle segment, *a* and *b* were fixed, thus producing a constant trajectory for all trials and participants (with *a*_*1*_ = 37*, a*_*2*_ = −3*, a*_*3*_ = 26 and *b*_*1*_ = 23, *b*_*2*_ = −15, *b*_*3*_ = −9). The first and third segments of the target trajectory were generated randomly for each trial (see Fig. 1). To create smooth transitions between adjacent segments, gaps of 30 data points were inserted and interpolated via cubic splines. The target velocity was held constant along the trajectory. Three different velocity levels were implemented by varying the distance between the trajectory coordinates spaced equally along the trajectory. The participants were instructed to continuously track the target, but they were not informed that one trajectory segment was repeated across all trials. In each trial, the target started in the screen’s center and moved on an invisible trajectory to the left or right at one of three velocity levels, resulting in six conditions (low, medium or high velocity × right or left side) that were counterbalanced across all trials. There were 12 repeats of the six respective experimental conditions, resulting in 72 trials with a break after every 10th trial to prevent fatigue effects.

The participants controlled only vertical movements of the cursor; the horizontal movement was fixed to the target. The mean trial duration was 12 seconds, resulting in an average task duration of 20 minutes. The coordinates of cursor and target were sampled with 60 Hz, equal to the framerate of the screen.

### Calculation of the temporal tracking error

**Fig. 2:**
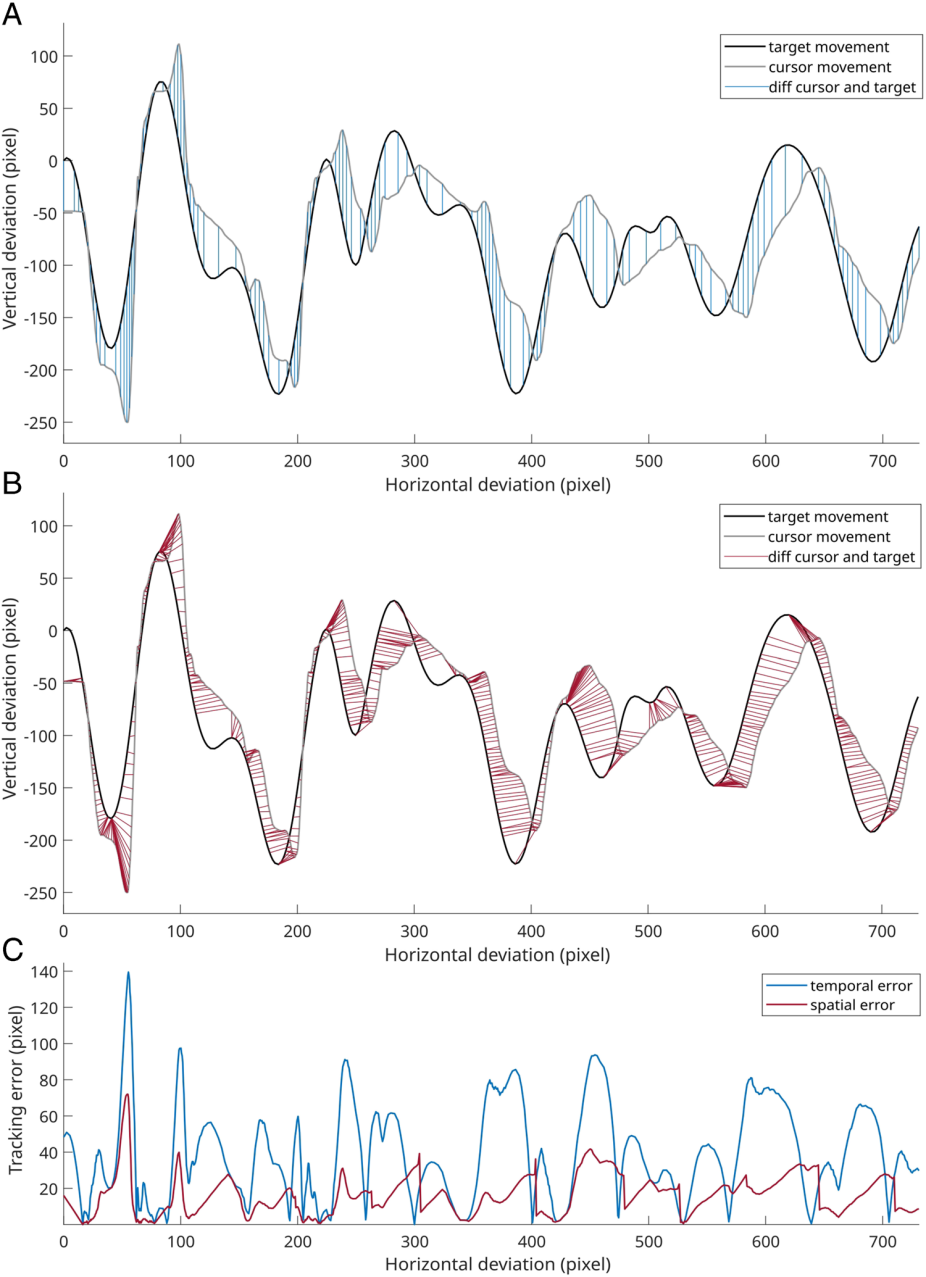
Values considered for the calculation of temporal and spatial tracking error and their result. **A** The blue lines depict the difference between cursor and target position, which is then used for calculating the temporal tracking error. The blue lines therefore indicate time points and are depicted for every fifth sample. **B** The red lines show the distance between the cursor position and the respective tracked target position as identified by the algorithm presented herein. **C** The resulting temporal (blue) and spatial (red) tracking error for the trial shown in (**A** and **B**)

The temporal tracking error is defined as the deviation of the cursor and target at time point *t* projected on the y-axis because the horizontal motion of the cursor is fixed to the target. Note that the temporal error is not an error in time, but the difference of cursor and target based on the temporal allocation of coordinates. For an entire trial, the temporal error would be the mean vertical deviation of the cursor and target over all frames in time in this trial. This is commonly known as root mean square error (RMSE). As this tracking error is based on simultaneously comparing the cursor and target position, it is called a temporal tracking error. As described in the introduction, this error measure suffers from weaknesses that lead to limited interpretability. For instance, the error is minimal when the trajectories of the cursor and target overlap, which does not necessarily indicate good tracking performance but may be caused by a change of target direction unexpected by the participant (see Fig. 2A, C).

### Calculation of the spatial tracking error

We differentiate the trajectory coordinate shown with a particular cursor sample at time point *t* (i.e., the temporal error) and the putatively aimed trajectory coordinate of a subject at time point *t.* The latter is the *intended* trajectory coordinate*.* While the temporal tracking error is insufficient to quantify the distance to the intended trajectory at a particular time point or cursor coordinate, the spatial error considers the intended trajectory at time point *t*. It not only quantifies the difference of cursor and target in the vertical dimension but also provides a measure of the distance in 2D space (i.e., considering both vertical and horizontal dimensions) to the intended trajectory positions (see Fig. 2B).

#### Assumptions for estimating the intended trajectory

To calculate the distance to the intended trajectory coordinates (i.e., the spatial error), we must identify the intended or aimed target trajectory sample for each cursor pursuit sample. That is because during the pursuit-tracking task, participants might not necessarily try to keep the cursor close to the target they see but instead follow the target trajectory with some latency or partly anticipate the upcoming target trajectory. These more complex cognitive processes cannot be accounted for by the temporal error and require calculating the spatial error of cursor pursuit and intended target trajectory. To find the intended target position (hereafter referred to as the trajectory sample) for each cursor position (hereafter, pursuit sample), we make two main assumptions: (1) If a cursor movement is intended to track a specific target movement, these movements should be similar to each other, *i.e., assumption of similarity*. For instance, if the cursor changes the direction from moving upwards to moving downwards, the intended trajectory part is most probably a direction change from upwards to downwards as well. (2) The intended target movement is temporally and spatially close to the cursor movement, *i.e., the assumption of proximity*. Considering the direction change example, this indicates that the intended trajectory sample can be found in the closest similar target direction change to the respective cursor direction change. In the following, we propose a procedure for estimating the intended trajectory for every pursuit sample based on the assumptions mentioned above.

#### The TRACK algorithm

In pursuit tracking, a change of direction of the cursor can be regarded as an active decision in response to a perceived change of target direction. Hence, we use directional changes in cursor and target movement to identify the intended target trajectory as anchoring events. To find all time points of direction changes of target and cursor, we identify the local maxima and minima of the trajectory and the pursuit. This is done by identifying all samples that are either larger (local maximum) or smaller (minimum) than their two neighboring samples, i.e., direction changes within 50 ms. Since the target trajectory is generated by concatenating sine and cosine terms, all maxima and minima are considered for the analysis. This is different for the cursor pursuit because steering the joystick provokes smaller fluctuations in the signal, which are not intended changes in direction. The minimum prominence for peak (maxima and minima) detection in the cursor pursuit is set to 20 pixels to increase the chance of selecting only the intended direction changes. Prominence, in this case, describes how distinct the peak is compared to other peaks regarding its height and location. For other screen sizes, we recommend to adjust this parameter accordingly, so that it is set to approximately 2% of the screen size.

After identifying all direction changes of cursor and target, the intended target direction change for each cursor direction change is estimated. This estimation is based on the two assumptions described earlier: First, the direction changes are similar and close in the temporal and spatial domains. The similarity criterion is given by only selecting target minima for cursor minima and target maxima for cursor maxima. Second, the proximity of the cursor and target movements is given by calculating a weighted distance measure of the cursor and target coordinates, where the distance of the two points on the x-axis is weighted double to ensure temporal proximity of the events.

**Fig. 3:**
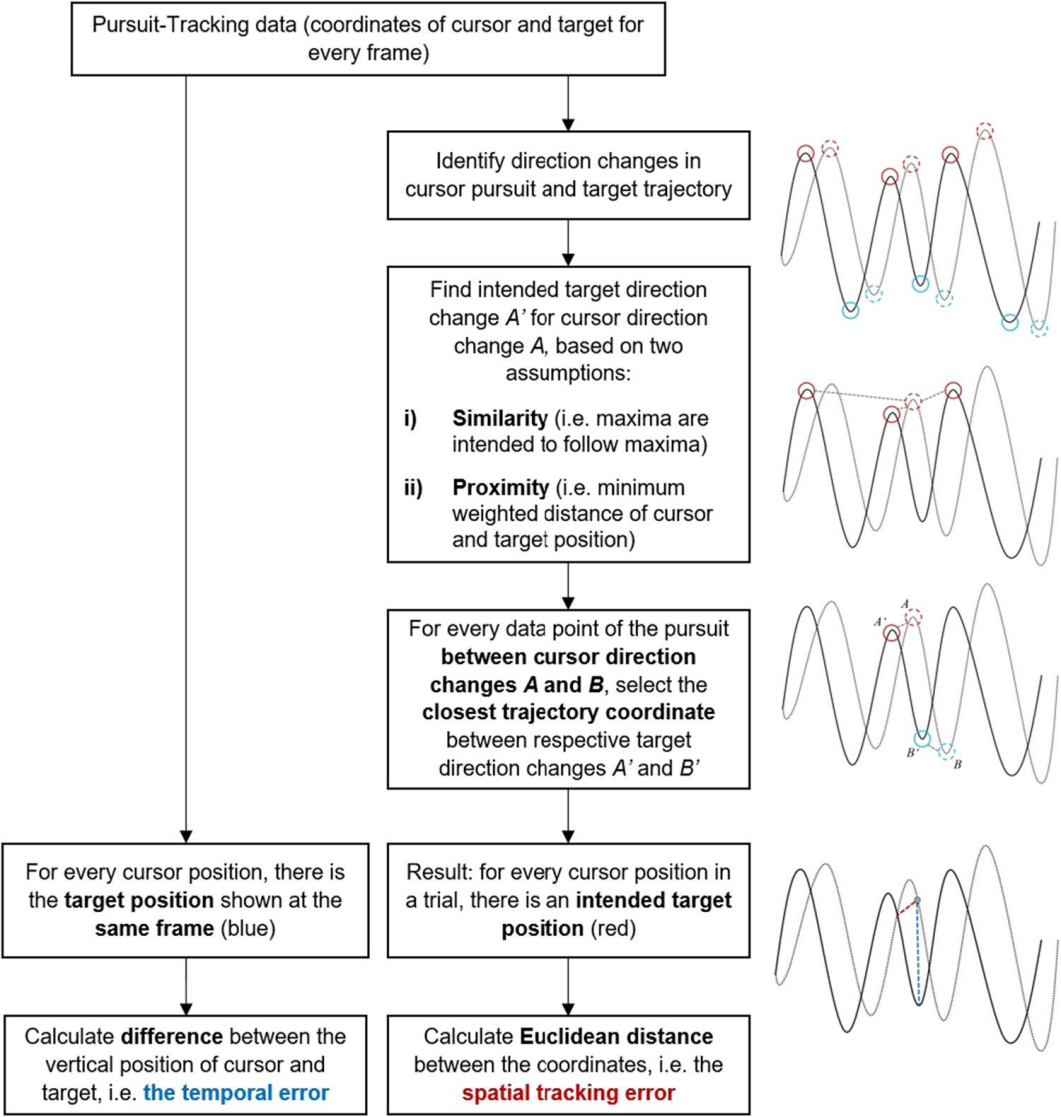
Overview of the steps needed to calculate the temporal and the spatial tracking error

After all maxima and minima of the pursuit have been assigned to matching maxima and minima of the target trajectory, the intended trajectory samples between these direction changes are determined. This is done in two steps. First, for each pursuit sample, the surrounding maximum and minimum and the matching trajectory maximum and minimum are determined. As the pursuit sample is enclosed by the maximum and minimum, the intended trajectory is also enclosed by the corresponding maximum and minimum. Therefore, all trajectory samples enclosed by the matching trajectory direction changes are “candidates” for the intended trajectory of this pursuit sample. Second, the closest sample of these candidates is then determined as the intended trajectory sample for this pursuit sample. All pursuit values between a maximum *A* and a minimum *B* are assumed to be anticipations or tracking the trajectory samples between the respective trajectory maximum *A*′ and minimum *B*′. Following this approach, the intended trajectory for every pursuit sample can be derived from the data. Given the pursuit coordinates and the respective intended target coordinates, the spatial error is calculated as Euclidean distance *d* between the two points. For an overview of the whole “TRACK” algorithm, see Fig. 3.

Since the spatial error measure provides information on the spatial, i.e., vertical, as well as temporal, i.e., horizontal, dimension of the tracking performance, it enables the classification of adaptive and anticipatory cursor movements. That is, if the intended trajectory sample appears temporally after the respective pursuit sample, i.e., if the x-position of the intended trajectory sample is larger than the x-position of the pursuit sample, it is labeled as anticipative because a target direction change was predicted and executed prematurely.

### Participants/sample

To evaluate and validate the performance of the algorithm, we applied it to empirical data from *N* < 34 healthy volunteers performing a pursuit-tracking task. One participant was excluded due to incomplete data recording, resulting in a sample of *N* < 33 participants from age 20 to 30 (25.26 ± 2.96 years). There were 11 male and 22 female participants; all were right-handed. All participants reported no history of psychiatric or neurological illness in the past six months, and all had normal or corrected-to-normal vision. All participants provided written informed consent and were compensated for their participation. The university’s ethics committee approved the study, and the study was conducted in accordance with the Declaration of Helsinki. For demonstration purposes, we provide tracking data for 10 trials of two participants, respectively.

### Apparatus

The pursuit-tracking task was displayed on a 32″ screen (Asus VG248QE) with a resolution of 1920×1080 pixels and a refresh rate of 60 Hz. Participants sat in front of the screen with a viewing distance of approximately 60 cm. The target was a red square and the cursor displayed a white cross. Both stimuli had a diameter of 0.79° visual angle (32×32 pixels) and were shown on a black background. Participants operated a joystick (Thrustmaster T.16000M) with their right hand to move the cursor. They controlled only vertical movements of the cursor; the horizontal movement was tied to the target. More precisely, there was a 1:1 mapping of joystick deflection and cursor movement in the vertical direction, whereas moving the joystick horizontally did not affect the cursor. Full joystick deflection moved the cursor to the respective vertical screen border, relaxing the joystick put the cursor in the center of the screen. The target movement was computed by a self-developed program using PsychoPy2 (Peirce et al., 2019).

### Exclusion of erroneous trials

Trials are labeled as erroneous if there was a deviation of more than 100 pixels from the center of the screen at the start of a trial, indicating that the joystick was not relaxed, thus producing an error peak. Furthermore, trials are labeled as potentially erroneous if the highest temporal error in the trial exceeds three standard deviations above the mean highest error across all subjects and trials. To ensure that only trials with erroneous, i.e., random, tracking behavior and not those with bad tracking performance are excluded, visual checks of every potentially erroneous trial are performed before excluding any trial.

### Validation of the spatial tracking error and statistical analysis

For every included trial, the mean temporal and spatial tracking error is calculated for the repeated and random trajectory segments. Both random segments are averaged as mean error for the random trajectory. Note that the first and third segment, i.e., both random segments, in our empirical data differ significantly regarding tracking performance, likely due to an intra-trial fatigue. However, we assume that an average of both segments approximates a fatigue level comparable to the constant, i.e., middle trajectory segment.

Further, the rate of anticipated intended target coordinates is calculated for each trial and trajectory segment as the ratio of anticipated samples/overall number of samples. All trials are retrospectively grouped into three equal blocks to investigate the development of the temporal and spatial tracking error across the experiment. Since direction changes in target and cursor movements are the key event for allocating target and cursor coordinates, we investigate differences in the mean number of cursor (with a minimum prominence of 20 pixels) and target direction changes through a *t*-test.

First, to differentiate temporal and spatial tracking error, we calculate a 2×3×3 repeated-measures analysis of variance (ANOVA) (based on the general linear model) for the mean tracking error with the within-subject factors error type (spatial/temporal), trial block (first, second or last trial block) and trajectory segment (repeated or averaged random segment). Second, to further investigate the validity of the spatial tracking error, we conduct another repeated-measures ANOVA for the spatial tracking error with the within-subject factors anticipation (anticipated/adapted intended target coordinate), trial block and trajectory segment, resulting in a 2×3×2 design. Then, for validating the measure of anticipation, we calculate a 2×3 repeated-measures ANOVA with the within-subject factors trajectory segment and trial block. A Greenhouse–Geisser correction is applied if necessary. Effect sizes are given for all significant (*p* <.05) results (Cohen’s *d*_*z*_ for *t*-tests and *ƞ*^*2*^_*p*_ for *F*-tests). For exploratory analysis of the distribution of temporal and spatial tracking error, we visually inspect the distribution of both errors across the constant, i.e., repeated, trajectory segment. All error values are given in pixels. For descriptive statistics, we provide the mean and standard error. Confidence intervals in Fig. 6 are adjusted following the procedure described by Loftus and Masson (1994).

## Results

### Estimation of intended trajectory samples

**Fig. 4:**
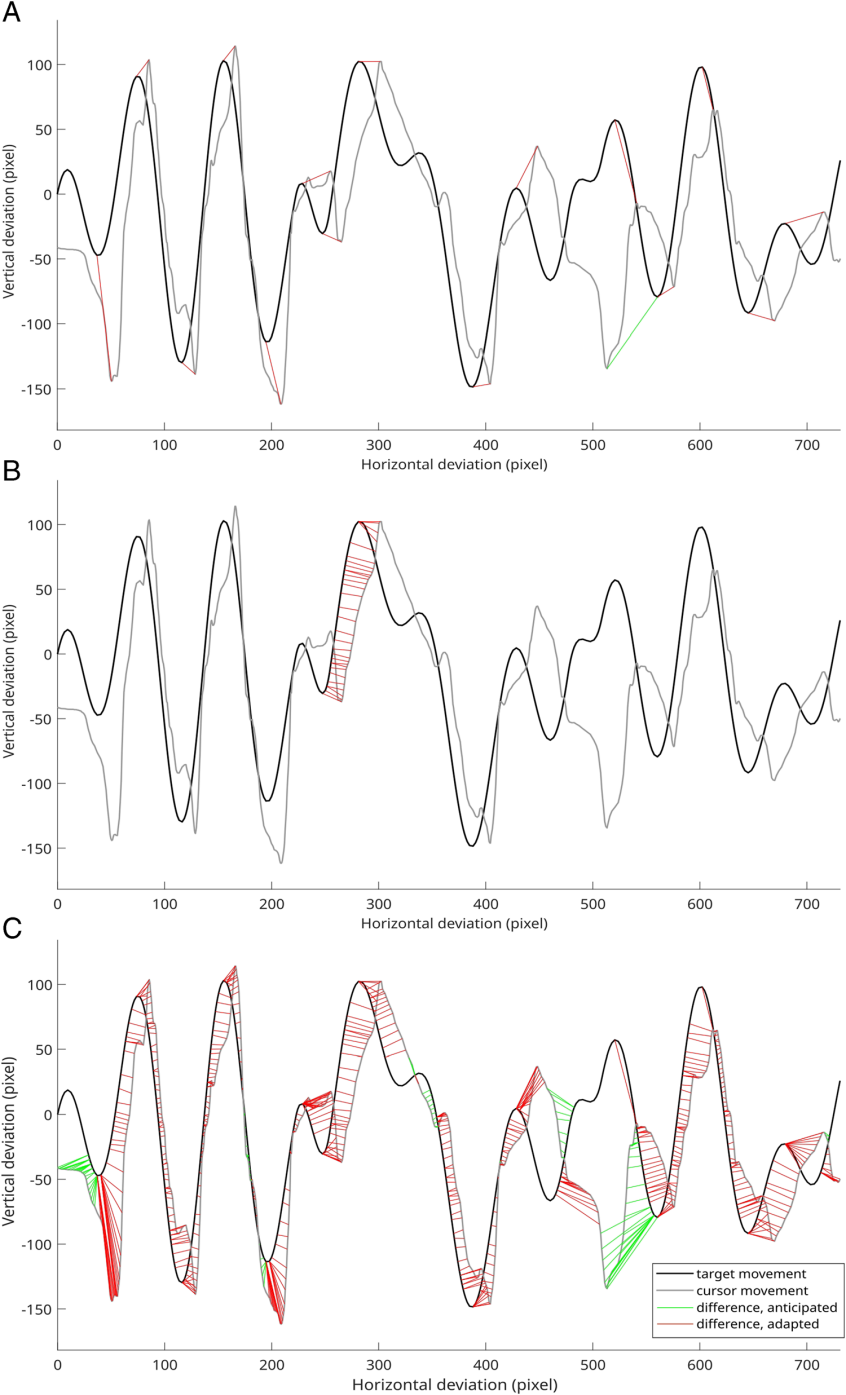
Steps for identifying intended trajectory samples shown for one exemplary trial. Target trajectory depicted as black line, cursor pursuit as grey line. **A** Matching maxima and minima are connected by red or green lines. If the cursor direction change occurs before the target direction change, the cursor direction change is labeled as anticipatory and the direction changes are connected by a green line. Otherwise, the direction change is labeled as adaptive and the connecting line is displayed in red. **B** Intended trajectory samples for pursuit samples between one minimum and maximum. **C** Intended trajectory samples for all pursuit samples. Anticipatory values are connected by green lines, adaptive values by red lines

Intended trajectory coordinates were estimated for pursuit coordinates following the described procedures. One exemplary allocation process for one trial is depicted in Fig. 4. An intended trajectory coordinate could be estimated for every pursuit coordinate.

**Fig. 5:**
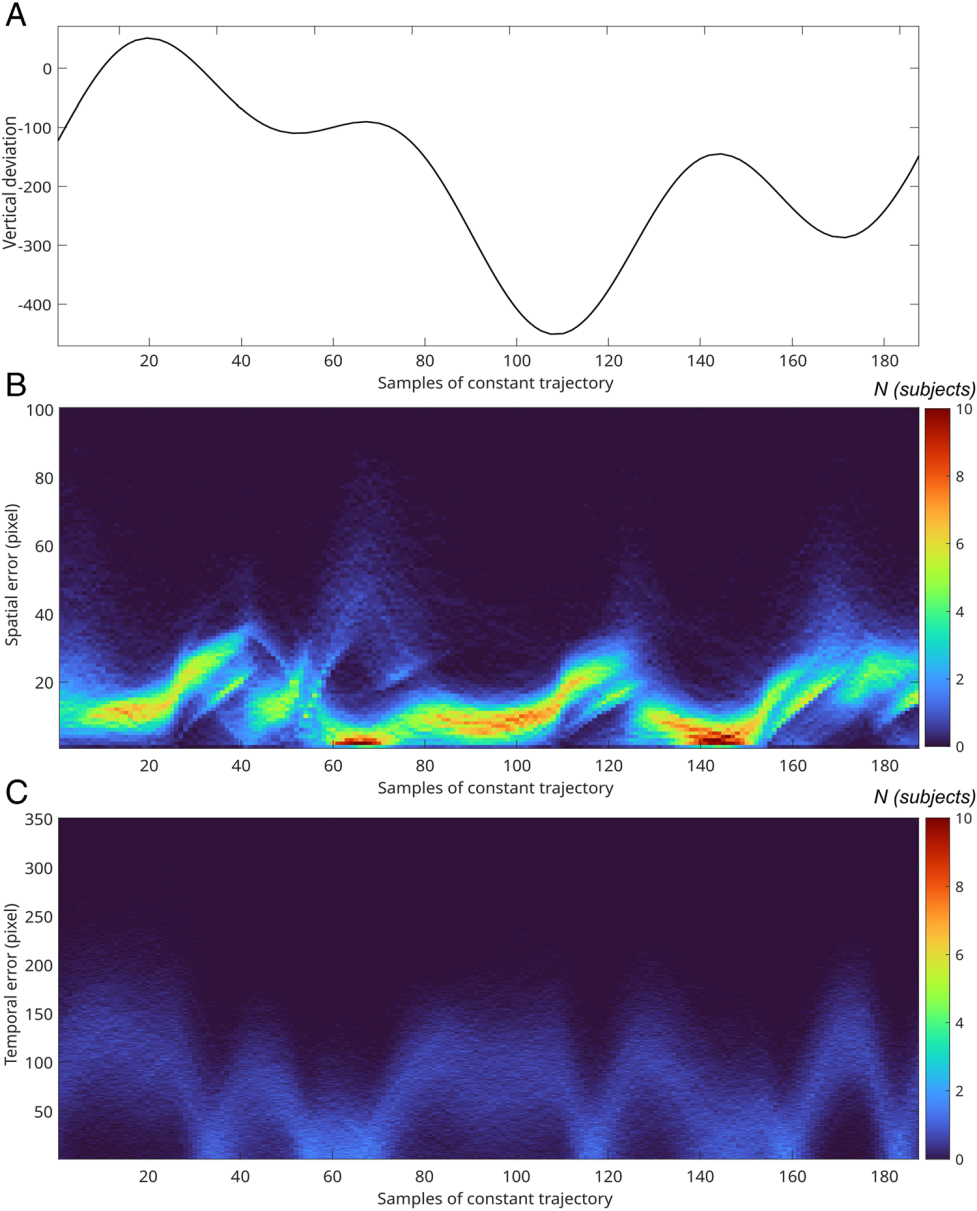
**A** The repeated trajectory segment. **B** The distribution of the spatial error for each sample of the repeated trajectory. Vertical lines depict the error distribution for that sample of the repeated trajectory. The frequency, i.e., number of subjects with a certain average error value for this sample, is depicted by color. **C** The distribution of the temporal error for each sample of the repeated trajectory. For instance, at the 100th sample of the repeated trajectory, the spatial error appears to be distributed around a mean of approximately 10 pixels, whereas the temporal error shows a broader distribution around a mean of approximately 120 pixels

A *t*-test revealed a significantly smaller number of direction changes of the cursor (15.988 ± 0.526) compared to the target (19.267 ± 0.187) with *t*(32) = 33.167, *p* < .001, *d*_*z*_ = 5.774. The distribution of temporal and spatial error along the samples of the constant trajectory is depicted in Fig. 5. The error distribution correlates visibly with the course of the repeated trajectory for both error types. However, there are visible differences between the two approaches. For instance, the temporal error has a minimum approximately at sample 115 (see Fig. 5C), while the spatial error increases (see Fig. 5B). Note that the trajectory path at this time point (see Fig. 5A) shows a target direction change shortly before, which is most often followed by a crossing of cursor and target, when the cursor movement direction is adjusted to the changed direction of target movement. Another difference is that the temporal error exhibits broad unimodal error distributions at each sample of the constant trajectory, whereas the spatial error can detect complex distributions. However, this was not tested statistically.

### Differentiation of temporal and spatial tracking error

**Fig. 6:**
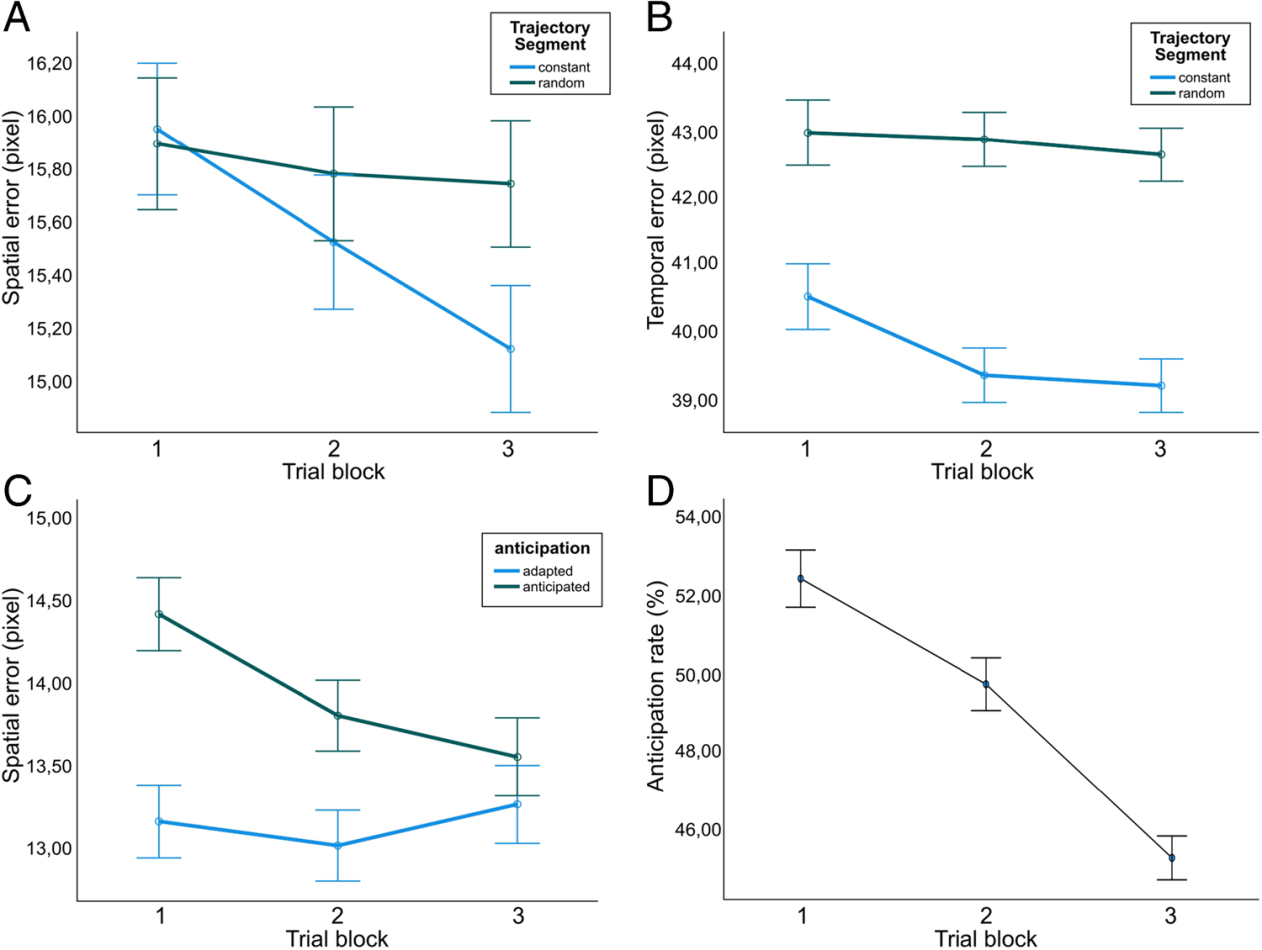
Means and adjusted confidence intervals based on the procedure of Loftus and Masson (1994). **A** Interaction effect of trial block and trajectory segment for the spatial error data. **B** Interaction effect of trial block and trajectory segment for the temporal error data. **C** Interaction effect of anticipation and trial block for the spatial error data. **D** The anticipation rate across trial blocks

Descriptive data of the behavioral performance in the tracking task are shown in Fig. 6. Figure 6A and B depict the spatial and temporal error for all trial blocks and both trajectory segments. In Fig. 6C, the spatial error for all three trial blocks is contrasted for adapted and anticipated trajectory coordinates. Figure 6D shows the anticipation rate contrasted for all trial blocks.

The repeated-measures ANOVA of the tracking error showed a significant main effect for error type, with higher values for the temporal error (41.105 ± 0.662) than for the spatial error (15.678 ± 0.309), with *F*(1,32) *=* 3825.78, *p <* .001, *ƞ*^*2*^_*p*_ = 0.992. Furthermore, there was a significant main effect of the trajectory segment with larger tracking error in the averaged random trajectory (29.237 ± 0.474) compared to the constant trajectory (27.546 ± 0.511), with *F*(1,32) = 39.02, *p <* .001 and *ƞ*^*2*^_*p*_ = 0.549. There was a significant main effect of the trial block, with significantly larger errors in the first trial block (28.761 ± 0.479) compared to the third trial block (28.105 ± 0.484), but no significant differences to the middle trial block (28.309 ± 0.495), *F*(2,64) = 6.346, *p =* 0.003 and *ƞ*^*2*^_*p*_ = 0.166.

We found a significant interaction effect between error type and trajectory segment with *F*(1,32) = 165.287, *p <* .001 and *ƞ*^*2*^_*p*_ = 0.838. Post hoc *t*-tests showed significant differences between the trajectory segments (constant segment: 39.55 ± 0.714; averaged random segment: 42.659 ± 0.657) for the temporal error (*t(*32) = 8.699, *p <* .001, *d*_*z*_ = −1.514), but not for the spatial error (*t(*32) = 1.326, *p* = 0.097).

Furthermore, there was a significant interaction of trajectory segment and trial block with *F*(2,64) *=* 5.386, *p =* 0.007 and *ƞ*^*2*^_*p*_ = 0.144. Post hoc ANOVAs revealed a significant effect of the trial block for the constant trajectory segment with a mean tracking error of 28.169 ± 0.535 for the first trial block, 27.374 ± 0.547 for the second trial block and 27.097 ± 0.507 for the last trial block (*F*(2,64) *=* 10.541, *p <* .001, *ƞ*^*2*^_*p*_ = 0.248), but no significant effect of the trial block for the mean tracking error in the averaged random trajectory segment (*F*(2,64) *=* 0.587, *p =* 0.559).

There was no significant interaction of error type and trial block (*F*(2,64) *=* 1.639, *p =* 0.202). Further, the interaction of error type, trial block and trajectory segment was not significant (*F*(2,64) *=* 2.909, *p =* 0.062).

### Validation of the spatial tracking error

For the spatial tracking error, there was a significant main effect of anticipation (13.157 ± 0.356 for adaptive segments; 13.935 ± 0.352 for anticipated segments) with *F*(1,32) *=* 28.996, *p <* .001, *ƞ*^*2*^_*p*_ = 0.475. Furthermore, there was a significant main effect for trial block with a higher spatial error in the first trials (13.8 ± 0.364) compared to the second third of the trials (13.417 ± 0.387) and the last trials (13.420 ± 0.322) with *F*(2,64) = 3.653, *p =* 0.031, *ƞ*^*2*^_*p*_ = 0.102. Also, the trajectory segment showed a significant main effect for the spatial error, with a smaller spatial error for the constant trajectory part (13.094 ± 0.362) compared to the averaged random trajectory (13.998 ± 0.364), *F*(1,32) *=* 17.168, *p <* .001 and *ƞ*^*2*^_*p*_ = 0.349.

There was a significant interaction effect of anticipation and trial block, with *F*(2,64) *=* 5.651, *p =* 0.005 and *ƞ*^*2*^ = 0.150. In post hoc analyses, a significant main effect of trial block for anticipated segments was found (*F*(2,64) *=* 7.852, *p <* .001, *ƞ*^*2*^_*p*_ = 0.197), but not for adapted segments (*F*(2, 64) *=* 0.733, *p =* 0.484). For the anticipated segments, the mean spatial error was highest in the first trial block (14.428 ± 0.372), smaller for the middle trial block (13.811 ± 0.417) and smallest for the last trial block (13.565 ± 0.330).

There was no significant interaction of trial block and trajectory segment (*F*(2,64) *=* 2.904, *p =* 0.062) and no significant interaction effect of anticipation and trajectory segment (*F*(2,64) *=* 0.646, *p =* 0.428). The interaction of anticipation, trial block and trajectory segment also showed no significant result (*F*(2,64) *=* 0.041, *p =* 0.960).

### Validation of the anticipation measure

For the rate of anticipated target coordinates in the spatial tracking error, we found a significant main effect of trial block (*F*(2,64) *=* 132.617, *p <* .001, *ƞ*^*2*^_*p*_ = 0.806) with a mean anticipation rate of 0.525 ± 0.004 for the first trial block, 0.498 ± 0.003 for the second trial block and 0.453 ± 0.003 for the third trial block. Neither the main effect of the trajectory segment (*F*(1,32) *=* 0.17, *p =* 0.897), nor the interaction of trial block and trajectory segment (*F*(2,64) *=* 0.108, *p =* 0.898) were significant.

## Discussion

The pursuit-tracking task provides an experimental paradigm to assess complex sensorimotor integration processes and continuously measure performance monitoring with a higher ecological validity than simple tasks. While there is a need for accurate measures of tracking performance, the classical tracking error does not provide the full information contained in the continuous data, since it often only relies on the vertical difference between cursor and target. To circumvent the limited interpretability resulting from this measure and use the full potential of the task, we developed a new error measure, the spatial tracking error, by accounting for the target position that was intended at every cursor position. We provide an algorithm to estimate the intended target coordinate for every data point of the pursuit and a script to calculate the temporal, i.e., classical, and spatial tracking error. Using the algorithm, we estimated the intended target position based on the assumptions of proximity and similarity between direction changes of cursor and target. The algorithm is open source (https://osf.io/stz7u/) and can be used for continuous pursuit-tracking data where a cursor is supposed to follow a target moving on a trigonometric trajectory.

Our empirical data comparing spatial and temporal tracking error showed that the tracking performance improved over the experiment irrespective of the error type and that there was a smaller error for the repeated trajectory segment. The mean tracking error improved mainly in the repeated segment, and no practice effect was found for the random segment. This indicates that participants improve their tracking performance by implicitly learning the repeated trajectory segment, while they do not improve in spontaneously tracking an unknown trajectory, replicating a robust finding in the pursuit-tracking literature (Wulf & Shea, 2002). Importantly, there was a smaller spatial tracking error compared to the temporal or “classical” measure. Since the tracking error calculated through different approaches can be viewed as a composite of behavioral and model-based error, an overall smaller spatial tracking error suggests that this measure is potentially able to explain more variance in tracking behavior.

Investigating influences on the spatial error, our empirical data show a practice effect, i.e., a decrease in the spatial tracking error across the experiment. Further, the spatial error was smaller in the repeated segment, showing that the measure can detect the implicit learning effect. We found a higher spatial tracking error in the segments labeled as “anticipated” by our algorithm compared to those labeled as “adapted”. Furthermore, the spatial error specifically decreased for the anticipated values, but not in the adapted parts, over the course of the experiment. The anticipation rate was higher in earlier trials, but did not differ for the repeated and random trajectory parts. Since there is an improvement in tracking performance over time, together with a decrease in the anticipation rate, the participants seem to learn to track the trajectory but without being able to anticipate it. This is congruent with the findings for the trajectory segment: also here, there is a better performance in the repeated segment but there is no higher anticipation rate for this segment. An improvement in tracking performance without an increase in the anticipation rate could be explained by the fact that the assumption that better tracking is associated with anticipation of the trajectory is inaccurate. However, another explanation could be that the measure of anticipation is so far still prone to error.

Yet, our new measure and algorithm for tracking performance can replicate established effects of the pursuit-tracking paradigm, such as the implicit learning of the repeated trajectory segment (Ewolds et al., 2017, 2021; Künzell et al., 2016; Wulf & Schmidt, 1997). Importantly, this shows that the spatial error is a valid measure for the tracking error. Beyond its ability to replicate established findings, we found indications that the spatial error provides new diagnostic aspects in the measurement of continuous tracking performance. The spatial error appears less diffuse and noisy than the temporal error, thus providing a sharper quantification of the tracking error. Furthermore, the interpretive issues of the temporal error, i.e., the minimal error at random target and cursor crossings, are not present for the temporal error (see Fig. 5).

## Limitations

We infer the tracking error from both vertical and horizontal distance to the target, whereas the horizontal distance to the target is inferred from the intended target position as identified by our algorithm. In addition, other distance measures could be applied as well, which can be further investigated in future development of the algorithm. Moreover, the identification of intended target positions is mainly based on direction changes of cursor and target. However, this crucially depends on the number of direction changes of both cursor and target. If there are more direction changes of the cursor than performed by the target, this can make the algorithm more prone to errors.

## Conclusion

The pursuit-tracking paradigm gives the opportunity to gain a more naturalistic insight into complex sensorimotor integration processes. However, the established error measure for tracking performance has some weaknesses that limit its interpretability. We developed an algorithm to calculate a new error measure, the spatial tracking error, derived from the distance to the intended target position. We can identify the intended target position based on the assumptions of proximity and similarity of target and cursor direction changes. By applying our algorithm to pursuit-tracking data, we found that the spatial error replicates established effects of the pursuit-tracking task. Beyond replication of established findings, we showed that the spatial tracking error fits tracking behavior better and provides novel insights and parameters for research utilizing tracking tasks. Our method provides an important step for fully exploiting the potential of pursuit-track tasks for research on sensorimotor integration processes.

## Data Availability

Relevant data and material is available at https://osf.io/stz7u/.
